# A paradox of immunodeficiency and inflammation in human aging: lessons learned from apoptosis

**DOI:** 10.1186/1742-4933-3-5

**Published:** 2006-05-19

**Authors:** Sudhir Gupta, Anshu Agrawal, Sudhanshu Agrawal, Houfen Su, Sastry Gollapudi

**Affiliations:** 1 Laboratories of Cellular and Molecular Immunology, Division of Basic and Clinical Immunology, University of California, Irvine, California 92697, USA

## Abstract

Aging is associated with a paradox of immunodeficiency and inflammation (an evidence of hyperactive immune system). Apoptosis is associated with cellular depletion and suppression of inflammatory response. In this brief review, we will present evidence for the role of increased apoptosis in immunodeficiency and paradoxical increased inflammation associated with human aging. In particular, a role of apoptotic cells in failure to generate anti-inflammatory responses and directly activating inflammatory responses will be discussed.

## Introduction

Aging represents a paradox of immune deficiency and chronic inflammation. Immune deficiency is predominantly associated with progressive decline in T cell functions in both mice and humans and numbers in humans [1-11], whereas, chronic inflammation is evidenced by increased circulating levels of pro-inflammatory cytokines, (IL-6, TNF-α, IL-1β,), acute phase proteins including C-reactive protein and serum amyloid A, and increased frequency of chronic inflammatory diseases of aging such as Alzheimer's Parkinson's diseases, amyotrophic lateral sclerosis, atherosclerosis etc. [12-19].

Apoptosis is a physiological form of programmed cell death which plays an important role in cellular homeostasis in the immune system in the selection of T cell repertoire, deletion of self-reactive lymphocytes, and cytotoxic response against target cells [20]. One of the features of apoptosis is that the cell death is associated with lack of inflammatory response (c.f. necrosis). Apoptosis is associated with blebbing of plasma membrane and the development of apoptotic bodies, which contain nuclear and cytoplasmic contents that are readily taken up by neighboring phagocytic cells. Apoptotic bodies inhibit inflammatory response of phagocytic cells by inducing anti-inflammatory cytokines [21,22]. We and others have demonstrated that human T cells and T cell subsets from aged humans display increased sensitivity to death receptor-induced apoptosis [23-28], which may play an important role in T cell deficiency associated with aging. In disease states, including aging which is associated with increased apoptosis we would expect that increased apoptotic bodies would be taken-up by the neighboring phagocytic cells and inhibit pro-inflammatory response. However, aging is associated with chronic inflammatory state. In this review we will discuss a role of increased apoptosis of various subsets of CD4+ and CD8+ T cells in immunodeficiency associated with aging and the role of apoptotic bodies in failure to suppress pro-inflammatory response in dendritic cells as one of the mechanisms of chronic inflammation associated with human aging.

## Apoptosis

**Figure 1:**
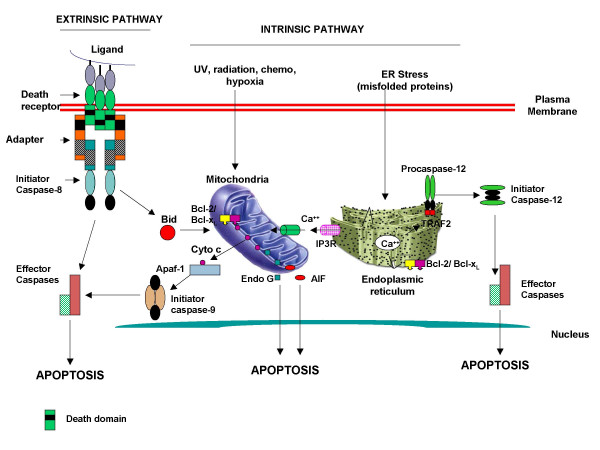
Death receptor and intrinsic pathways of apoptosis. Intrinsic pathway is mediated by mitochondrial and the endoplasmic reticulum pathways. Distinct initiator caspases are activated in each pathway of apoptosis (modified from ref. 94).

Apoptosis is mediated by two major pathways, the extrinsic or death receptor-mediated pathway and the intrinsic pathway, which is mediated via mitochondria and the endoplasmic reticulum (Figure 1; [29-34]). In aging, death receptor pathway of apoptosis has been extensively examined; however, mitochondrial and the endoreticulum pathways of apoptosis in aging have not been studied in detail. Therefore, we will focus our discussion on death receptor-mediated apoptosis in human aging. Death receptors belong to a large family of tumor necrosis factor receptors (TNFRs). Following interaction with death receptor ligand, the cytoplasmic death domains (DD) of death receptors undergo trimerization. Since cytoplasmic domains of death receptors lack enzymatic activity they recruit a set of adaptor proteins by protein-protein interaction and proximal or initiator caspases (caspase-8, caspase-10) forming a death-inducing signaling complex (DISC). Caspases are present in an inactivated prozyme form. In the DISC, initiator caspases are activated by homodimerization and without undergoing cleavage and are released from the disc into the cytoplasm where they serve as enzymes for effector pro-caspases (caspase-3, caspase-6, and caspase-7). Initiator caspases cleave effector pro-caspase to generate active effector caspase, which cleave a large number of cytoplasmic and nuclear substrates to induce morphological and biochemical features of apoptosis [31]. Among cell death receptors CD95- and TNFR-mediated apoptosis has been extensively studies.

CD95 is constitutively expressed on a subset of T cells and is upregulated following activation; CD95L is lacking on resting T cells and is induced upon activation. CD95L is also proteolytically cleaved and may be present in the soluble form in the serum. Interaction between CD95 and soluble CD95L or anti-CD95 antibody results in trimerization of cytoplasmic death domain of CD95 [20,29]. CD95 DD recruits an adaptor protein, the fas-associated death domain (FADD), which contain a death effector domain. FADD then recruits procaspase-8 to form DISC. Pro-caspase-8 is autolytically activated and is released into the cytoplasm where it activates effector caspases (a point of no return) to induced apoptosis.

**Figure 2:**
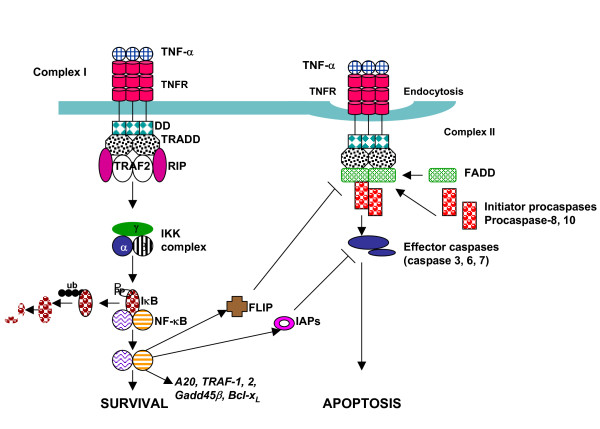
TNF receptor (TNFR) pathway of signaling. Two complex model is shown. Upon ligation with TNF-α (with in 10–20 min), TNFR I undergo trimerization and recruits various adapter molecules resulting in the activation of NF-κB, which induces several anti-apoptotic genes (Complex I formation) and survival signal. This is followed by (more than 2–3 hours) by an endocytosis of receptor complex resulting in the dissociation of certain adapter proteins (TRAF-2, RIP) and recruitment of fas – associated death domain (FADD) and procasepase-8 to form death-inducing signaling complex (DISC). In the DISC, caspase-8 is activated and released into the cytoplasm where it activates effector caspases to induce apoptosis.

TNF-α induces signaling via both TNFR-1 and TNFR-2. TNFR-1 contains DD and induces both survival and cell death signals, whereas TNFR-2 lack cytoplasmic DD and predominantly provides survival signal; however, may enhance apoptosis-mediated by TNFR-1 [34-38]. Recently, Tschopps and his associated have suggested two complex model for TNF-α-induced activation of NF-κB (Figure 2), which provides a survival signal and caspase activation and also provides an apoptotic signal [39]. In this model, an interaction between TNF-α and TNFR-1, results in trimerization of DD of TNFR-I, which in turn recruits an adaptor protein, the TNFR-associated death domain (TRADD). TRADD then recruits TNFR-associated factor-2 (TRAF-2) and (receptor interacting protein (RIP) forming signaling complex I (within minutes), which results in the activation of NF-κB. Signaling complex 1 activates NF-κB via recruitment of IκB kinase (IKK) complex and phosphorylation of IκB, which is a signal for ubiquitination and subsequent proteasomal degradation of IκB resulting in the release of NF-κB and its translocation and binding to DNA to induce production of a number of anti-apoptotic proteins [40-43]. The signaling complex 2 is formed possibly following TNFR-1 internalization (>2 hours following interaction between TNF-α and TNFR-1), resulting in the dissociation from RIP, and TRAF-2 from TNFR-1and recruitment of FADD and caspase-8 forming a DISC and finally activation of effector caspases and induction of apoptosis. When NF-κB activation is strong, anti-apoptotic proteins inhibit activation of casapse activation in complex II; however, a weak complex I signaling results in weak or deficient NF-κB activation. As a result the products of anti-apoptotic genes are not made (at least in normal quantity) and complex II can signal apoptosis via activation of caspases. There is an evidence that TRAF-2 also activate JNK-2 (via activation of MEKK1), which cleaves Bid into jBid (distinct from tBid, which is a product of caspase-8 cleavage). jBid translocates to the mitochondria and preferentially releases Smac/Diablo from the mitochondria, which may disrupt TRAF-2/cIAP1 (cellular Inhibitor of Apoptosis Protein 1) complex formation and inhibition of caspase activation [44,45]. In addition, Diablo inhibits anti-apoptotic effects of cIAP and XIAP by binding to them.

## Apoptosis of T cell subsets in aging

Unlike mice, human aging is associated with lymphopenia which is shared by both CD4+ and CD8+ T cells [46,47], which may play a role in increased frequency of infections and malignancies in aging. We and others have reported increased apoptosis in T cells, CD4+ and CD8+ T cells in human aging. These have been recently reviewed and readers are referred to recent reviews [25,48].

Several investigators have reported increased sensitivity of T cells from aged humans to activation-induced cell death (AICD), which is mediated via CD95-CD95L interaction [26,27]. We have observed increased sensitivity of both CD4+ and CD8+ T cells from aged humans to CD95-mediated apoptosis, which is associated with increased activation of caspase-8 and caspase-3 [23,49]. Furthermore, we observed increased expression of FADD.

We have examined TNF-α-induced apoptosis in T cells and T cell subsets in aging. Previously we have reported that both CD4+ and CD8+ T cells from aged humans display increased sensitivity to TNF-α-induced apoptosis [24]. Furthermore, we demonstrated that signaling downstream of TNFR-1 was involved [25]. We showed that increased sensitivity of aged T cells to TNF-α-induced apoptosis was associated with decreased NF-κB activation due to decreased phosphorylation of both IKKβ and IκB [50], decreased expression of cIAPs [51], and increased expression of FADD [52]. Overexpression of dominant negative FADD in aged T ells resulted in normalization of apoptosis to the level observed in T cells from young subjects. Similarly overexpression of IKK resulted in upregulation of cIAP, increased phosphorylation of IκB, and inhibition of apoptosis in aged T cells to a level observed with young T cells, suggesting that decreased NF-κB plays an important role in increased sensitivity of aged T cells to apoptosis [50,51]. Several other investigators have also reported decreased NF-κB activation in T cells in aging [53,54].

More recently, we have analyzed TNF-α-induced apoptosis in naïve and different memory T cells. Following activation with an antigen, naïve T cells undergo clonal expansion and following clearance of antigen majority of antigen-specific T cells are removed by apoptosis and a small pool of antigen-specific T cells are retained as memory T cell pool. Based upon their homing characteristics, cytokine production, and effector functions memory T cells have been further subdivided into central memory (T_CM_, which localized to lymph nodes and demonstrate high replicative potentials) and effector memory (T_EM_, which are localized in non-lymphoid tissues and display poor proliferative potential) T cells [55-58]. These subsets are identified by the presence and absence of a set of cell surface markers. CD8+ effector memory T cells are further subdivided into two subsets T_EM _(CD45RA-) and T_EMRA _(CD45RA+), whereas CD4+ effector memory cells are primarily T_EM _and only 1–2% are T_EMRA_; however, they are increased in aging [48]. In human aging, the number of T_N _and T_CM _CD8+ T cells is significantly reduced, whereas the numbers of T_EMRA _CD8+ T cells is increased [59-62]. We have shown that naïve (T_N_) and T_CM _CD8+ T cells are sensitive to TNF-α-induced apoptosis, whereas T_EM _and T_EMRA _CD8+ T cells are relatively resistant to apoptosis. Furthermore, T_N _and T_CM _CD8+ T cells from aged humans are significantly more sensitive to TNF-α-induced apoptosis as compared to those from young subjects, which may be responsible, at least in part, for decreased T_N _and T_CM _in aging [63]. However, no significant difference was observed in TNF-α-induced apoptosis in T_EM _or T_EMRA _CD8+ T cells between young and aged subjects. We have observed that T_EMRA _CD8+ T cells preferentially proliferate in response to IL-15 as compared to naïve and TCM CD8+ T cells, which proliferate preferentially in response to IL-7 (unpublished observations). Furthermore, in our gene array analysis of CD8+ T cells from aged ad young subjects by Affymatrix we have observed upregulation of IL-15 gene in CD8+ T cells in aged humans (unpublished observation). This would suggest that the accumulation of T_EMRA _CD8+ T cells in aging is not due to changes in apoptosis and may be due to increased growth secondary to increased IL-15 in aging. Berard et al [64] have reported that IL-15 promotes survival of naïve and memory CD8+ T cells (based upon the expression of CD44) in mice. However, these investigators did not characterized memory CD8+ T cells into T_CM _and T_EM _cells. Dunne et al [65] reported that following acute Epstein-Barr virus infection, CD45RA+ CD8+ T cells (most likely T_EMRA_, although these investigators did not use other markers to confirm that these cells are indeed T_EMRA_) proliferate in response to IL-15. These investigators did not compare the effect of IL-15 on T_CM _and T_N _CD8+ T cells.

**Figure 3:**
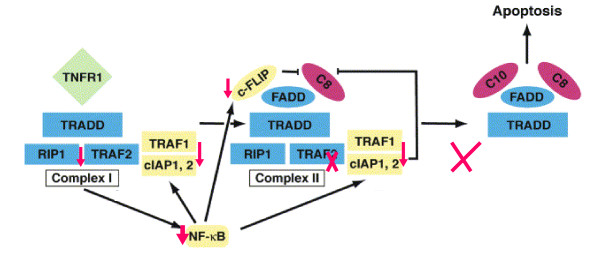
Proposed mechanisms of increased sensitivity of aged T_N _and T_CM _CD8+ T cells to TNF-α-induced apoptosis. Signaling molecules downstream of TNFRs that activate NF-κB are decreased in aging resulting in decreased NF-κB activity and decreased expression of anti-apoptotic proteins.

We have investigated the molecular basis of increased sensitivity of T_N_and T_CM _CD8+ T cells from aging subjects to TNF-α-induced apoptosis. TNFR-1 and TNFR-2 expression on T_N _and T_CM>_CD8+ T cells is similar between young and aged subjects [63], suggesting that downstream signaling events are likely responsible for increase sensitivity to apoptosis. Therefore, we have compared downstream signaling molecules in TNFR signaling pathway between young and aged humans (unpublished data). TNF-α-induced NF-κB activation in T_N _and T_CM _CD8+ T cells in aging is reduced as compared to young subjects. Furthermore, we observed that TNF-α-induced JNK activation of reduced, suggesting that TNFR-mediated mitochondrial pathway (via activation of jBid) is unlikely responsible for increased sensitivity to apoptosis. Since activation of IKK complex and phosphorylation of IκB are necessary for the activation of NF-κB [42,43], we examined TNF-α-induced phosphorylation of IKKα/β and of IκB. CD8+CD28+ T cells (T_N _plus T_CM_) from aged subjects had significantly lower phosphorylation of both IKKα/β and IκB as compared to young controls. IKK complex is activated by TRAF-2 via RIP and NIK [66-68]. In aged CD8+CD28+ T cells the expression of TRAF-2, RIP, and NIK was decreased as compared to young subjects. NF-κB mediates its survival signaling by inducing production of anti-apoptotic proteins, including Gadd45β, Bcl-x_L_, A20, IAPs, and FLIP [69,70]. Gadd45β inhibits apoptosis by inhibiting JNK activation [71]. Furthermore, Bcl-x_L _regulates apoptosis by inhibiting mitochondrial pathway of apoptosis. Since in aged T_N _and T_CM _CD8+ T cells JNK activation is decreased, it is unlikely that mitochondrial pathway of apoptosis and therefore, Gadd45β and Bcl-x_L _play a significant role increased sensitivity to TNF-α-induced apoptosis. We have observed decreased expression of cIAP1, FLIP, and A20 in T_N _and T_CM _CD8+ T cells in aged subjects as compared to young subjects. Which of these NF-κB target anti-apoptotic genes play a role in increased sensitivity of T_N _and T_CM _CD8+ T cells in aging remains to be determined? A similar pattern of apoptosis though not as striking as in CD8+ subsets has been observed with CD4+ T cells [48]. A model of proposed mechanisms of increased TNF-α-induced apoptosis in T_N _and T_CM _CD8+ T cells in aging is shown in Figure 3.

## Role of apoptotic cells in the regulation of inflammation and changes in aging

Apoptotic cell death and clearance of dead cells is of vital importance in developing and maintaining the normal tissue homeostasis and resolution of inflammation. The most remarkable aspect of the process of cell death is the targeted elimination of apoptotic cells without inflammation or pathology. There is growing evidence that the clearance of apoptotic cells by phagocytosis can result in anti-inflammatory and immunosuppressive effects [72]. This is supported by the observations that the defective clearance of apoptosis is associated with autoimmunity and inflammation [73]. Co-culture of apoptotic cells with macrophages results in an active suppression of proinflammatory cytokines, including TNF-α, whereas the production of anti-inflammatory transforming growth factor-β (TGF-β) and IL-10 is increased [21,22]. Further studies have revealed that TGF-β1, PGE2 and platelet activating factor (PAF) all have paracrine/autocrine role in inhibiting TNF-α secretion [74]. Cells that have undergone apoptosis without being phagocytosed undergo secondary necrosis, releasing some of its contents, including heat shock proteins, which interact with Toll-like receptors on antigen presenting cells (APC) to induce inflammatory cytokines. In addition, apoptotic cells can directly induce caspase-1-mediated release of pro-inflammatory IL-1 and IL-8 from the dying cells [75,76].

In summary, under normal conditions, clearance of apoptotic cells by phagocytic cells is associated with secretion of anti-inflammatory cytokines, including IL-10 and TGF-β1 resulting in the inhibition of inflammation. However, under pathological conditions associated with excessive apoptosis and/or decreased clearance of apoptotic cells, apoptotic cells may directly induce caspase-1 dependent secretion of IL-1β and IL-8 or undergo secondary necrosis to induce secretion of other pro-inflammatory cytokines, including TNF-α by macrophages via release of endogenous ligands (e.g. heat shock proteins) for TLR.

Dendritic cells (DCs) are the professional antigen presenting phagocytes. Immature dendritic cells are capable of large scale phagocytosis of apoptotic cells by a mechanism that involve bridging of thrombospondin 1 (TSP1) and DC integrins and CD36 [77,78] The maturation of immature DCs by LPS and other stimuli can be inhibited by engulfment of apoptotic cells as evidenced by the suppressed upregulation of key co-stimulatory molecule CD86 [79-82] and the reduced expression of IL-12 by DCs. DCs on stimulation with LPS in the presence of apoptotic cells secrete decreased amounts of TNF-α and IL-12 [79-81]. However, in contrast to macrophages TGF-β and IL-10 production by DCs is not increased following ingestion of apoptotic cells. Therefore, phagocytosis of apoptotic cells by DCs inhibits secretion of pro-inflammatory cytokines by an unknown mechanism, which is independent of TGF-β1 and IL-10. The maturation state of DCs acts as a checkpoint in the initiation of immunity and inflammation. Immature DCs are highly phagocytic, express low levels of MHC and co-stimulatory molecules and do not produce inflammatory cytokines. Maturation of DCs is associated with downregulation of phagocytic capacity, upregulation of MHC and co-stimulatory molecules, and secretion of pro-inflammatory cytokines.

Aging maybe considered a "chronic inflammatory" condition as evidenced by elevated levels of circulatory pro-inflammatory cytokines, including IL-6, TNF-α, PGE-3, and anti-inflammatory mediators, such as IL-1 receptor antagonists, soluble TNFRs and acute phase proteins, in elderly subjects [17,83] However, the mechanisms underlying this chronic inflammatory state and increased pro-inflammatory cytokines in aging in presently unclear. Several studies regarding a role of macrophages and T cells in elevated levels of pro-inflammatory cytokines in aging has produced conflicting data [17]. We have investigated a role of DCs, especially in relation to uptake of apoptotic cells and regulation of inflammatory response in aging. We have observed that DCs from aged humans display a more mature phenotype (increased MHC and co-stimulatory molecule), decreased capacity to phagocytose apoptotic lymphocytes, and increased production of pro-inflammatory cytokines TNF-α and IL-6 as compared to DCs from young subjects (unpublished data). Unlike DCs from young subjects, co-culture of apoptotic cells with LPS-stimulated DCs from aged subjects failed to Inhibit secretion of IL-6 and TNF-α, therefore contributing to an increased pro-inflammatory cytokine production by DCs from aged subjects. Furthermore, we have observed that LPS-stimulated DCs from aged subjects in the absence of apoptotic cells also secrete higher levels of IL-6 and TNF-α as compared to young subjects. Inefficient clearance of apoptotic cells by aged DCs may lead to extracellular accumulation of apoptotic cells and subsequent secondary necrosis of apoptotic cells, which may induce maturation and activation of DCs via production of endogenous danger signals (e.g. heat shock protein) to induce pro-inflammatory cytokine production. Furthermore, extracellular apoptotic cells may also trigger caspase-1-mediated secretion of IL-1β and IL-18 from dying cells. Mature phenotype DCs in aged subjects may sample self antigens from extracellularly accumulated apoptotic cells and from those undergone secondary necrosis to induce autoimmune response. Therefore, more mature phenotype of DCs and inefficient clearance of apoptotic cells may result in both chronic inflammation and autoimmunity, two features commonly observed in aging

Therefore, we propose that apoptosis plays an important role in the pathogenesis of chronic inflammation during human aging by two mechanisms, [1] a defective clearance of apoptotic cells as a result of poor phagocytosis of apoptotic cells by aged DCs results in secondary necrosis and release of endogenous ligands for TLRs to activate DCs to differentiate into more mature phenotype and secrete pro-inflammatory cytokines (e.g. TNF-α and IL-6) and [2] increased number of apoptotic lymphocytes in aged humans may directly trigger caspase-1-mediated IL-1β and IL-8 release from dying cells.
